# Knowledge, attitudes, and practices of active surveillance in prostate cancer among urologists: a real-life survey from Brazil

**DOI:** 10.1186/s12894-022-01036-1

**Published:** 2022-06-15

**Authors:** Marcelo Langer Wroclawski, Breno Santos Amaral, Paulo Priante Kayano, Wilson Francisco Schreiner Busato, Sebastião José Westphal, Erik Montagna, Bianca Bianco, Andrey Soares, Fernando Cotait Maluf, Gustavo Caserta Lemos, Arie Carneiro

**Affiliations:** 1 Hospital Israelita Albert Einstein, Rua Iguatemi, 192, cj. 43, São Paulo, SP CEP: 04530-050 Brazil; 2 BP - A Beneficência Portuguesa de São Paulo, São Paulo, SP Brazil; 3 Faculdade de Medicina do ABC, Santo Andre, SP Brazil; 4 Universidade do Vale do Itajaí – UNIVALI, Itajaí, SC Brazil; 5 Sociedade Brasileira de Urologia, Rio de Janeiro, RJ Brazil; 6 Centro Paulista de Oncologia - Oncoclínicas, São Paulo, SP Brazil; 7 Latin American Cooperative Oncology Group - Genitourinary, Porto Alegre, RS Brazil

**Keywords:** Active surveillance, Prostate cancer, Survey

## Abstract

**Background:**

Active surveillance (AS) is the preferred treatment for patients with very low-and low-risk prostate cancer (PCa), but it is underperformed worldwide. This study aimed to report knowledge, attitudes, and practices (KAP) of AS for PCa among urologists in Brazil.

**Methods:**

This cross-sectional study used a questionnaire with 50 questions divided into participant characteristics, knowledge regarding inclusion criteria for AS, follow-up, intervention triggers, acceptance, and practice for an index patient. Data analysis comprises absolute and relative frequencies of the variables. After that, a logistic regression was performed in order to verify possible patterns of answers provided by the respondents in the index patient questionnaire.

**Results:**

Questionnaires were sent through the SurveyMonkey® platform to 5,015 urologists using email addresses and through social media. A total of 600 (12%) questionnaires returned and 413 (8.2%) were completed and included in the analysis. Only 53% of urologists adopt AS for low- and very-low-risk PCa. Inclusion criteria were patients with age > 50 years (32.2%), prostate specific antigen (PSA) < 10 ng/mL (87.2%), T1 clinical stage (80.4%), Biopsy Gleason score ≤ 6, positive cores ≤ 2 (44.3%), positive core involvement < 50% (45.3%), and magnetic resonance imaging findings (38.7%). The PSA doubling time was still used by 60.3%. Confirmatory biopsy (55.9%), PSA level (36.6%), and digital rectal examination (34.4%) were considered by most urologists for follow-ups. Patient preference (85.7%), upgrade of Gleason score (73.4%), and increased number of positive cores (66.8%) were associated with conversion to definitive treatment. In an index patient, non-acceptance and active treatment request were the most cited reasons for not performing AS.

**Conclusion:**

There is significant variability in the KAP of AS in Brazil, which indicates the need to reinforce AS, its inclusion and follow-up criteria, and the benefits for physicians and the general population.

*Trial registration***:** Not applicable.

**Supplementary Information:**

The online version contains supplementary material available at 10.1186/s12894-022-01036-1.

## Background

There were approximately 65,000 New cases of prostate cancer (PCa) in Brazil in 2020. In 2017, mortality due to neoplasia was 15,576 [1]. In the last two decades, PCa diagnosis has increased, especially in the low-risk category, mainly because of more widespread prostate specific antigen (PSA) screening [2].

Active surveillance (AS) is a safe option for low- and very low-risk PCa [3, 4]. Current guidelines from the European Association of Urology (EAU) [5], American Urological Association (AUA) [6], and National Comprehensive Cancer Network (NCCN) [7] recommend AS as the preferred treatment option for these patients. Intermediate-risk PCa patients present with poor outcomes when managed through AS, but its use should be considered in selected cases. Nevertheless, AS remains underperformed worldwide [8].

This study aimed to report the knowledge, attitudes, and practices (KAP) of AS for PCa among urologists in Brazil.

## Methods

This cross-sectional study followed a checklist for reporting the E-Survey results [9].

An anonymous survey was created to assess KAP of AS for PCa among urologists in Brazil. Questionnaires were sent through the SurveyMonkey® platform to 5015 urologists from the Brazilian urological community using email addresses obtained from the Brazilian Society of Urology membership directory and through social media.

The instrument for data collection was developed ad hoc for this study and comprised two parts consisting in the mapping of fundamental concepts in AS and the validation of the categories identified. The first part adopted a delphi approach through semi-structured interviews until saturation, that is, when little or no variation is observed in the answers [10]. Participants were specialists in Urologic oncology. The interview consisted of three questions: (1) List the topics or knowledge that a doctor should already know about AS; (2) What are the typical and exclusive contents of AS? (3) List the most common misconceptions about AS. The common points mentioned were aggregated by equality, similarity or thematic coherence in order to reduce the number of conceptual categories. This procedure was carried out independently by two researchers with ties dismissed by a third participant. The saturation point was reached after three interviews, as the answers were similar, equivalent or complementary [11].

The survey consisted of 50 questions, divided into participant characteristics (five items), acceptance and inclusion criteria to select patients for AS (11 items), practice characteristics (nine items), follow-up protocol (five items), intervention triggers (11 items), and knowledge about index patient (65-year-old male without comorbidities, potent and continent, diagnosed with very low or low-risk PCa) (nine items) (Additional file 1).

The inclusion criteria included being a Brazilian urologist and signing an informed consent. Incomplete questionnaires were discarded.

The sample size for a population of unknown size, with non-random distribution, a 95% confidence level, and a 5% margin of error, required 385 respondents for a survey.

The data analysis pipeline comprised descriptive statistics steps in terms of the absolute and relative frequencies of the study variables. Categorical variables were compared using the chi-squared test. Pairwise comparisons were performed for all variables. Then, a chi-squared test of independence was used, where classes of respondents were the dependent variable and tested against items of the index patient knowledge part of the questionnaire. A logistic regression was performed in order to verify possible patterns of answers provided by the respondents in the “index patient knowledge questionnaire”. Considering that the variables of interest are categorical and that the analysis was based on the occurrence of responses instead of a yes/no response ratio, a binomial distribution was adopted for the logit (link function), with a non-robust covariance. The regression results were presented as non-adjusted regression coefficients (R). Statistical significance was set at *p* < 0.05. Statistical analyses were performed using the R Studio version 1.1.383.

## Results

### Participants characteristics

Questionnaires were sent to 5015 urologists. A total of 600 (600/5015, 12%) questionnaires returned and 413 (413/5015, 8.2%) were completed and included in the analysis. The characteristics of the respondents are listed in Table 1.

**Table 1:** Characteristics of the participants

Variables		N	%
*Age (years)*^a^			
45.8	SD 11.2 (27–74)	–	–
*Years of professional activity*^a^			
16.6	SD 12.4 (0–52)	–	–
*Major type of workplace*			
Outpatient clinic or non-university hospital of the public health system		39	9.5
Exclusively Hospital and/or Private Clinic		131	31.7
50% Hospital and/or Private Clinic and 50% University-Hospital of the Public Health System		235	56.9
University Hospital of the Public Health System		8	1.9
*Continuing education*			
Residency in Urology		413	100
No other postgraduation course		298	72.1
Fellowship in Uro-oncology		52	12.6
Master degree		30	7.3
Ph.D.		33	8.0
*Region of Brazil*			
North		8	1.9
Northeast		63	15.3
Middle-west		30	7.3
Southeast		236	57.1
South		76	18.4

^a^Variables presented as mean, standard deviation (SD) and minimum and maximum values (0 stands for residents in training)

### Acceptance and Inclusion criteria to select patients for AS protocol

AS is the preferred treatment option for patients with low-risk and very low-risk PCa for 53% of urologists, while 5.3% do not recommend AS and consider any form of active treatment instead. Thirty percent of urologists reported that they did not use a predefined protocol for selecting patients who were candidates for AS. When a protocol was used (70% of the time), the one that was most frequently adopted was the one from John Hopkins (43.3%). Table 2 summarises the criteria considered for the AS indications.

**Table 2:** Acceptance and inclusion criteria to select patients for active surveillance protocol

Question	Items	N	%
*Acceptance*			
Do you adopt AS in your practice for patients with low-risk and very-low risk prostate cancer?	No	22	5.3
	Yes	219	53
	Occasionally	172	41.7
Do you adopt any protocol for AS?	No	124	30.0
	John Hopkins	179	43.3
	Toronto	82	19.9
	Other	28	6.8
*Inclusion criteria to select patients for AS protocol*			
Do you use any of the following items in your practice to define eligibility for active surveillance? *more than one answer per item was accepted			
Age of the patient (years)	Not informed	91	22.0
	> 50 years	133	32.2
	> 55 years	14	3.4
	> 60 years	93	22.5
	> 70 years	71	17.2
	> 80 years	11	2.7
PSA level (ng/mL)	Not considered	27	6.5
	< 10 ng/mL	360	87.2
	Up to15 ng/mL	12	2.9
	Up to 20 ng/mL	14	3.4
PSA density (ng/mL)	Not considered	220	53.3
	< 10 ng/mL	35	8.5
	< 15 ng/mL	130	31.5
	< 20 ng/mL	28	6.8
Doubling PSA time	Not considered	164	39.7
	> 6 months	54	13.1
	> 1 year	99	24.0
	> 2 years	66	16.0
	> 3 years	20	4.8
	> 4 years	10	2.4
Clinical staging	Not considered	15	3.6
	T1	332	80.4
	T2a	48	11.6
	T2b	11	2.7
	T2c	6	1.5
	T3	1	0.2
Gleason Score in the prostate biopsy	Not considered	4	1.0
	≤ 6	385	93.2
	7 (3 + 4) only in selected cases	22	5.3
	7 (4 + 3) only in selected cases	2	0.5
Number of positive cores	Not considered	26	6.3
	≤ 2 fragments	183	44.3
	≤ 3 fragments	137	33.2
	≤ 34% of the total fragments	41	9.9
	≤ 50% of the total fragments	26	6.3
Maximum involvement of each positive core	Not considered	47	11.4
	< 20%	127	30.8
	< 30%	52	12.6
	< 50%	187	45.3
Eligibility	Not considered	83	20.1
	MRI	160	38.7
	Slide review	25	6.1
	MRI e slide review	112	27.1
	Other	33	8.0

*AS* active surveillance, *MRI* magnetic resonance imaging, *PSA* prostate-specific antigen

Concerning age, 22.5% of urologists considered AS only for patients aged above 60 years and 17.2% indicated it only for patients aged above 70 years. A PSA level of < 10.0 ng/mL was an inclusion criterion for 87.2% of the urologists. More than half (53%) of the urologists did not consider PSA density (PSA-D) to suggest AS. In contrast, 60.3% used PSA doubling time (PSAdt) as an inclusion criterion for AS. For clinical staging indications, AS is an option for 80.4% of urologists in T1 tumours, 11.6% in T2a tumours, 2.7% in T2b tumours, and 1.5% in T2c tumours. A Gleason score (GS) of 6 or less was an inclusion criterion for 93.2% of the urologists and 5.3% considered AS for some selected Gleason 7 (3 + 4) cases, while 0.5% considered it for Gleason 7 (4 + 3) cases. Two or fewer positive cores were used by 44.3% of urologists, 33.2% used ≤ 3 positive cores, 9.9% used < 34% of the cores involved, and 6.3% did not use this as a criterion to indicate AS. Approximately 30.8% of urologists use < 20% as the maximum involvement of each positive core as a criterion to indicate AS, while 45.3% used 50% as the threshold to indicate AS. Approximately 38.7% of urologists use magnetic resonance imaging (MRI) as an eligibility criterion to allocate a patient to an AS protocol (Table 2).

### Practice characteristics

MRI is inaccessible to 15.7% of urologists, 9.2% have access to MRI of the pelvis, without a specific prostate protocol (multiparametric MRI (mpMR), and 1.5 and 3.0 Tesla mpMRI is available for 40.9% and 34.2% of urologists, respectively. Prostate biopsies are mostly performed at the same institution by the urologists (17.4%) who ordered it (17.4%), other urologists (19.6%), or radiologists (35.6%). The remaining 27.4% prostrate biopsies were performed elsewhere. The majority of biopsies were transrectal (99.3%). In terms of the number of cores retrieved, sextant biopsies (12 cores with two from each area) were the most common (41.6%). Fusion-guided biopsy was available for 52.3% of the urologists (23.7% with software, 28.6% cognitive), but 47.7% did not have access for their practice. The time interval between biopsy request and its execution was less than one month in most cases (67.1%). Biopsy histopathological reports provide GS in different ways. The vast majority provided Gleason for each specific fragment (87.9%). Occasionally, a unique GS is determined for fragments of the right lobe and another for fragments of the left lobe all together (5.6% of cases), and elsewhere only a single ‘general’ GS (6.5% of cases) is described. Histopathological reports do not indicate the number of positive cores and percentage of involvement of positive fragments in 1.7% and 3.6% of urologists’ practices, respectively (Table 3).

**Table 3:** Practice characteristics

Question	Items	N	%
*Practice characteristics*			
Considering the local where you practice most often			
Do patients have easy access to MRI?	No	65	15.7
	Multiparametric MRI 1.5 Tesla	169	40.9
	Multiparametric MRI 3.0 Tesla	141	34.2
	Pelvic (unspecified)	38	9.2
The prostate biopsy	It is performed at my service, by a urologist	81	19.6
	It is performed at my service, by a radiologist	147	35.6
	I do it myself	72	17.4
	It is outsourced	113	27.4
What is the access of prostate biopsy?	Transrectal	410	99.3
	Perineal	3	0.7
Number of cores retrieved	Sextant (6 cores)	4	1.0
	Sextant biopsies (12 cores with 2 of each area)	172	41.6
	12 distinct cores of the peripheral zone	57	13.8
	12 distinct cores of the peripheral zone plus 2–4 cores of the transition zone	26	7.3
	12 distinct cores of the peripheral zone plus 2–4 cores of the transition zone plus additional cores of suspected area (nodule in US or MRI)	142	34.4
	Other	12	2.9
Do you have access to biopsy with image fusion?	No	197	47.7
	Yes, with real-time software fusion	98	23.7
	Yes, with cognitive fusion	118	28.6
How long do patients have to wait for a biopsy to be performed?	Less than one month	277	67.1
	1 to 3 months	119	28.8
	3 to 6 months	15	3.6
	More than 6 months	2	0.5
Does the histopathological report of the prostate biopsy product provide the Gleason score?	Yes, of each fragment alone	363	87.9
	Yes, but a general Gleason Score	27	6.5
	Yes, for right lobe fragments and another one for left lobe fragments	23	5.6
Does the histopathological report of the prostate biopsy product specify how many fragments are positive?	No	7	1.7
	Yes	406	98.3
Does the histopathological report of the prostate biopsy product specify the percentage of tumor involvement in each fragment?	No	15	3.6
	Yes	398	96.4

*MRI* magnetic resonance imaging, *US* ultrasound

### Follow-up

During follow-up, 26.6% of urologists did not perform a confirmatory biopsy. The remaining patients underwent a confirmatory biopsy, mainly between 6 and 12 months after diagnosis (55.9%). Approximately 63% of urologists perform additional biopsies after a confirmatory biopsy, every one to two years (37.3% and 25.7%, respectively). The remaining only perform it if a trigger point is reached, such as a PSA rise (29.5%) or an MRI modification (7.5%) (Table 4).

**Table 4:** Follow-up protocol

Question	Items	N	%
*Follow-up protocol*			
In the follow-up of patients under AS:			
After the inclusion of a patient in an active surveillance protocol, do you perform a confirmatory biopsy?	No	110	26.6
	Yes, up to 3 months	18	4.4
	Yes, between 3 and 6 months	54	13.1
	Yes, between 6 and 12 months	231	55.9
In addition to the confirmatory biopsy, how often do you have new biopsies?	Yearly	154	37.3
	Biennial	106	25.7
	Only if PSA levels increase	122	29.5
	If there is a worsening in the imaging exam	31	7.5
In the first 5 years, how often do you evaluate PSA levels?	Quarterly	131	31.7
	Half-yearly	151	36.6
	Quarterly in the first two years and half-yearly between 2 and 5 years	117	28.3
	Annually	14	3.4
How often do you perform a digital rectal examination?	I do not use routinely	32	7.7
	Quarterly	41	9.9
	Quarterly in the first two years and half-yearly between 2 and 5 years	75	18.2
	Half-yearly measure	142	34.4
	Annually	123	29.8
In addition to ultrasound for biopsy, do you use other imaging tests?	No	93	22.5
	MRI occasionally	177	42.9
	MRI periodically	143	34.6

*AS* active surveillance, *MRI* magnetic resonance imaging, *PSA* prostate-specific antigen

### Intervention triggers

Triggers for active treatment during follow-up are presented in Table 5. More than one answer was permitted.

**Table 5:** Intervention trigger

Question	N	%
*Intervention trigger*		
Which of the following options do you use to recommend conversion from active surveillance to definitive treatment? *more than one answer was accepted		
Patient’s preference	354	85.7
Change in rectal examination (worsening of clinical staging)	250	60.5
Single rising PSA level (PSA > 10 ng/mL)	133	32.0
Two or more consecutive rising in PSA levels (PSA > 10 ng/mL)	225	54.5
PSA doubling time < 3 years	110	26.4
Rising PSA density (> 15%)	78	18.9
Gleason score upgrading to 7 (3 + 4)	228	55.2
Gleason score upgrading to 7 (3 + 4) or greater on re-biopsy	303	73.4
Increased tumor volume on imaging (MRI)	167	40.4
Higher number of positive cores in re-biopsy (> 1/3)	276	66.8
Involvement > 50% in at least 1 core in re-biopsy	189	45.8

*MRI* magnetic resonance imaging, *PSA* prostate-specific antigen

### Knowledge about index patient

The reasons for not performing AS in an index patient (65-year-old male without comorbidities, potent and continent, diagnosed with very low or low-risk PCa) are listed in Table 6. More than one answer was provided.

**Table 6:** Knowledge about index patient

Question	N	%
*Knowledge about index patient* *more than one answer was accepted		
Reasons for not performing active surveillance (AS) in an index-patient		
I don't know enough about this type of treatment to properly follow-up the patients	5	1.3
I believe that AS is an inappropriate approach for patients with prostate cancer, even at low or very-low risk	12	3.1
Morbidity associated with multiple biopsies	68	17.4
Patient does not accept the AS and requests active treatment	344	88.0
I would like to indicate, but the biopsy performed is inadequate	16	4.1
I would like to indicate, but I do not trust the team responsible for performing the biopsy	14	3.6
I would like to indicate, but the histopathological report does not provide the necessary data	17	4.3
I would like to indicate, but I do not trust the team responsible for the histopathological evaluation	20	5.1
I would like to indicate, but I cannot schedule laboratory tests and/or biopsy and/or outpatient return with the appropriate interval	42	10.7

### Response patterns

The logistic regression among ‘Indication of AS’ versus ‘Index Patient Knowledge’ was statistically significant for the following items: Considering those who did not indicate AS, there was a direct relation to ‘I don't know enough about this type of treatment to properly follow-up the patients’ (*p* = 0.030, R = 2.19) and ‘I believe that AS is an inappropriate approach for patients with prostate cancer, even at low or very-low risk’ (*p* = 0.004, R = 2.14), while an inverse relation was observed with ‘Patient does not accept AS and requests active treatment’ (*p* = 0.018, R = − 1.21). Considering those who indicate AS occasionally, there is a direct relation to ‘I would like to indicate, but I cannot schedule laboratory tests and/or biopsy and/or outpatient return with the appropriate interval’ (*p* = 0.026, R = 0.83), while an inverse relation was observed with ‘Morbidity associated with multiple biopsies’ (*p* = 0.001, R = -1.01) and ‘I would like to indicate, but the histopathological report does not provide the necessary data’ (*p* = 0.012, R = − 2.72). Finally, considering those who indicated AS, there was an inverse relation to ‘I believe that AS is an inappropriate approach for patients with PCa, even at low or very-low risk’ (*p* = 0.024, R = − 2.42), ‘Morbidity associated with multiple biopsies’ (*p* = 0.001, R = − 1.06), and ‘I would like to indicate, but I cannot schedule laboratory tests and/or biopsy and/or outpatient return with the appropriate interval’ (*p* = 0.048, R = − 0.74).

Regarding the region of Brazil and the indication of AS, multiple logistic regression showed a significant difference in the southern region (*p* = 0.028, R = 1.87).

## Discussion

AS has been proposed as the preferred treatment option for low- and very low-risk PCa; however, the inclusion criteria and follow-up schedules present great variability [3, 5, 12–17]. The features that vary most are the number of positive cores, proportion of cancer in each positive core, PSA cut-off, and use of PSA-D and PSAdt. The absence of homogeneous recommendations affects urological practice.

Total PSA up to 10 ng/dL is an inclusion criterion used by Klotz et al. [4], Tosoian et al. [3], Godtman et al. [12], and Bokhorst et al. [15]. Selvadurai et al. [13] accepted patients with PSA levels up to 15 ng/dL. Findings showed that the vast majority of urologists in Brazil use PSA < 10 ng/dL (87.2%) as the cut-off limit to propose AS.

Tosoian et al. [3] and Bokhorst et al. [15] considered PSA-D as an inclusion criterion. During follow-up, PSA-D is an important risk factor for progression of PCa [18]. In this study, up to 53.3% of urologists did not use it as an inclusion criterion. For those who adopt it, PSA-D < 15%, as in Johns Hopkins [3], is the cut-off for 31.5% and < 20%, as in PRIAS [15], is the cut-off for 6.8%.

In contrast, PSAdt has not been used by most of the AS protocols because this feature is a weak stage upgrading predictor in the specimen analysis after surgery [19]. Conversely, more than 60% of the responders still used PSAdt to indicate AS where 13.1% will offer AS if PSAdt is more than six months, and 24% will consider AS only if PSAdt is more than 12 months.

The number of positive cores and maximum tumour involvement in each core were both considered as inclusion criteria by Tosoian et al. [3], Welty et al. [20], Thomsen et al. [6], and Soloway et al. [7]. These characteristics are also linked to unfavourable evolution during follow-up and a higher chance of tumour reclassification [21]. The EAU [5] and NCCN guidelines [7] define very low-risk PCa when up to two cores are involved, as described by Epstein et al. [22]. However, AUA guidelines [6] classify very-low risk when there are < 34% positive cores, as the number of cores retrieved in a prostate biopsy has risen from the original six to a much greater sampling. In this study, 44.3% of urologists considered ≤ 2 positive cores, and 9.9% considered < 34% of the total cores as an inclusion criterion. A considerable number of urologists (33.2%) adopted a criterion of ≤ 3 positive cores, which was used by Thomsen [16].

Less than 50% maximum involvement of a positive core to include patients in an AS protocol was considered by 45.3% of the urologists, as used by Tosoian et al. [3], Welty et al. [20], and Thomsen et al. [16]. Notably, 43.4% of urologists used other cut-off limits as part of the inclusion criteria. Less than 20% of tumours in each core, as in Soloway et al. [17], was adopted by 30.8% urologists and less than 30% of tumours in each core, as in Thompson et al. [14], was used by 12.6%.

The follow-up management is also different in terms of the interval of digital rectal exam and PSA evaluation, and the role of confirmatory and re-biopsy indications varies among studies [3, 4, 13–17, 20]. This study also revealed similar differences in responses in terms of follow-up management preferences.

Although Brazil is a country with limited resources and a lack of MRI availability, a significant number of urologists order it to manage AS, even though its use during follow-up is still under investigation and has conflicting results [23, 24]. Also, it is has been demonstrated that MRI targeted biopsies can cause a “grade inflation” and this should be considered during the risk assessment [25]. The availability of MRI in Brazil varies according to the working environment (private versus public). In the public environment, mpMRI is accessible to 56.7% of urologists, and in almost two-thirds of these cases, only 1.5 Tesla equipment is available. In contrast, mpMRI can be easily ordered by 80% of urologists in private institutions, and half of them are 3 Tesla. The use of MRIs also varies according to geography, being less available in the northern and north-eastern regions, considered as economically disadvantaged areas compared to the rest of the country.

Since there is not a validated Brazilian guideline regarding the management of patients under AS, Brazilian urologists adapt their practice according to their own convenience, mixing criteria from diverse international guidelines, as demonstrated by our survey.

There was a direct relationship between the response of the urologists who did not indicate AS and the lack of knowledge about AS because they considered this type of treatment inappropriate for the index patient. While analysing those that indicated AS occasionally, we found that they did not consider the biopsy to be harmful and they have good pathology reports; however, they do not indicate AS regularly because of lack of access to scheduled laboratory tests and/or biopsy and/or outpatient return with the appropriate interval. As expected, urologists who indicated AS agreed to the harmfulness of biopsy were able to follow-up patients and had access to good pathology reports.

This study demonstrated that AS remains underused in Brazil. Only 53% of respondents considered it the preferred option for low- and very low-risk PCa in most cases. In developed countries, AS has gained traction in recent years. In the United States, up to 75% of very low-risk cases are initially treated with AS [26]. In Sweden, use of AS is even greater, reaching 91% for very-low-risk and 74% for low-risk cases [27].

Findings showed that economically less-favoured regions presented worse AS indications and acceptance rates when compared to more developed areas (South Region). This has also been observed in other studies. A survey demonstrated that AS is underused in Lebanon due to patient non-compliance and discrete acceptance among physicians [28]. Although the use of AS varies based on geographic location, county-level socioeconomic factors in the United States did not influence its indication [29].

Continuous medical education programmes and patient awareness campaigns are essential to reinforce AS and reduce the overtreatment of PCa and its potential complications. It is important to address the advantages of AS as a treatment option for the public, since the main reason for not performing AS was patient non-acceptance (88%).

This is the largest worldwide survey to evaluate AS and the first large-scale survey in low- to middle-income countries. Despite this, the response rate was 12% in the lower tier of what can be expected in voluntary e-mail-based surveys [30–32]. Another aspect that may represent a selection bias is that responders average age was 46.8 years old, maybe being more web/IT oriented, and prone to follow international guidelines and be updated to current practices.

We believe that this study described in detail Brazilian urologists’ knowledge, attitude, and practice regarding AS. Based on this data, public authorities and medical societies may have an instrument to guide the construction of a national guideline.

## Conclusions

PCa management through AS in a low-middle-income country presents many challenges. Many urologists do not adopt AS for a number of reasons. Continuous medical education and population awareness programmes are the most important methods to disseminate evidence-based knowledge in medical and general populations and to reduce overtreatment of PCa.

## Supplementary Information

**Additional file 1.** Survey about knowledge, acceptance, and practice of urologists concerning active surveillance for prostate cancer in Brazil.

## Data Availability

The datasets used and/or analyzed during the current study available from the corresponding author on reasonable request.

## Abbreviations

AS: Active surveillance
AUA: American Urological Association
EUA: European Association of Urology (EAU)
GS: Gleason score
KAP: Knowledge, attitudes, and practices (KAP)
mpMRI: Multiparametric magnetic resonance imaging
MRI: Magnetic resonance imaging
NCCN: National Comprehensive Cancer Network (NCCN)
PCa: Prostate cancer PC
PSA: Prostate specific antigen
PSA-D: PSA density
PSAdt: PSA doubling time
