# Supplementary material for: Knowledge, attitudes, and practices of active surveillance in prostate cancer among urologists: a real-life survey from Brazil
Source: BMC Urol. 2022 Jun 15;22:86. doi: 10.1186/s12894-022-01036-1 (PMC9199143; doi:10.1186/s12894-022-01036-1)
Supplement: Supplementary file 1 — Additional file 1. Survey about knowledge, acceptance, and practice of urologists concerning active surveillance for prostate cancer in Brazil. [file 12894_2022_1036_MOESM1_ESM.docx]

Additional file 1: English version of the questionnaire. Knowledge, attitudes, and practices of active surveillance in prostate cancer.

# Informed Consent Form

# By checking this field, you agree with the consent form above, considering that you have been informed of the objectives and relevance of the proposed study, how your participation will be, the procedures and risks arising from this study, and the authorisation that the data obtained in this research are used for scientific purposes (disclosure in events and publications).

# How old are you? (in years)

# Which state do you practice medicine in?

# Which municipality do you practice medicine in?

# What is your specialty?

# Options: 1) Urology, 2) Medical oncology, 3) Radiotherapy, and/or 4) Other (please specify).

# How long (in years) have you been practicing in this field (after your residency)?

# Do you have a urological oncology training?

# Options: 1) No; 2) Yes, fellowship; 3) Yes, Master’s degree; and/or 4) Yes, PhD.

# Where is your major type of workplace?

# Options: 1) Outpatient clinic or non-university hospital of the public health system, 2) exclusive hospital and/or private clinic, 3) >50% hospital and/or private clinic, 4) >50% University Hospital of the Public Health System, 5) Exclusively University Hospital of the Public Health System, or 6) > 50% Outpatient clinic or non-university hospital of the public health system.

1. How much of your practice is currently devoted to uro-oncology? (in %)
2. In the last year, approximately how many prostate cancer patients have you seen?
3. Among prostate cancer patients seen in the past year, how many are classified as low or very low risk? (in %)
4. Do you adopt active surveillance in your practice for patients with low-risk and very low-risk prostate cancer?

Options: 1) Yes, it's my first choice most of the time; 2) Occasionally, as an alternative to surgery/radiotherapy in selected cases; or 3) No, I always recommend active treatment.

1. Among the low-risk and very low-risk cases, what percentage of men have you indicated active surveillance in the past year?
2. Do you adopt any protocol for active surveillance?

Options: 1) No; 2) Yes, John Hopkins; 3) Yes, Toronto; or 4) Yes, Other (please specify)

1. If the patient meets the inclusion criteria for active surveillance, how is he usually advised? (More than one answer is acceptable).

Options: 1) By urologist, 2) By oncologist, 3) By the multidisciplinary team, 4) By specialist nurse through explanatory leaflet, and/or 5) Other (please specify)

1. After being counselled, does the male patient who accepts active surveillance need to sign an informed consent form?

Options: 1) Yes or 2) No

17. Do you use any of the following items in your practice to define eligibility for active surveillance? (More than one answer is acceptable).

Options: 1) Age, 2) PSA level, 3) PSA density, 4) PSA doubling time, 5) Clinical staging, 6) Gleason score in prostate biopsy, 7) Number of positive cores, 8) Maximum involvement of each positive core, and/or 9) Eligibility

18. Do you use any of the following items in your practice to define eligibility for active surveillance?

1) MRI, 2) Molecular biomarkers (OncoType Dx, Decipher or others), 3) Slide review, 4) None, and/or 5) Other (please specify)

19. At what age do you recommend active surveillance for men?

Options: 1) From 50 years old, 2) From 55 years old, 3) From 60 years old, 4) From 70 years old, 5) From 80 years old, or 9 ) I do not use this criterion to indicate active surveillance.

20. At what PSA value do you indicate need of active surveillance for patients? (More than one answer is acceptable).

Options: 1) Maximum of 10.0 ng/dL; 2) Up to 15.0 ng/dL; 3) Up to 20.0 ng/dL, in selected cases; and/or 4) I do not use this criterion to indicate active surveillance.

21. Which PSA density do you believe indicates active surveillance for patients?

Options: 1) <10%, 2) <15%, 3) <20%, or 4) I do not use this criterion to indicate active surveillance.

22. Which PSA doubling time do you indicate active surveillance for patients with?

Options: 1) >6 months, 2) >1 year, 3) >2 years, 4) >3 years, 5) >4 years, and 6) I do not use this criterion to indicate active surveillance.

23. At what clinical stage do you indicate active surveillance for patients? (More than one answer is acceptable).

Options: 1) T1, 2) T2a, 3) T2b, 4) T3, or 5) I do not use this criterion to indicate active surveillance.

24. What Gleason score(s) on biopsy do you indicate active surveillance for patients? (More than one answer is acceptable).

Options: 1) ≤6; 2) 7 (3+4), but only for selected cases; 3) 7 (4+3), but only for selected cases; and/or 4) I do not use this criterion to indicate active surveillance.

25. Consider the area where you practice most often. Does the pathology report of prostate biopsy provide the Gleason score?

Options: 1) Yes, of each fragment alone; 2) Yes, one Gleason for right lobe fragments and one for left lobe fragments; 3) Yes, but only a single ‘general’ Gleason score; or 4) No

26. Do you indicate active surveillance for men who have up to the following number of fragments affected in the biopsy?

Options: 1) two or fewer fragments, 2) three or fewer fragments, 3) less than 34% of the total fragments, 4) less than 50% of the total fragments, or 5) I do not use this criterion to indicate active surveillance.

27. Consider the area where you practice most often. Does the pathology report of prostate biopsy specify the number of fragments involved?

Options: 1) Yes or 2) No

28. Do you indicate active surveillance for patients with tumour involvement in each of the affected fragments?

Options: 1) <20%, 2) <30%, 3) <50%, or 4) I do not use this criterion to indicate active surveillance.

29. Consider the area where you practice most often. Does the pathology report of prostate biopsy specify the percentage of tumour involvement in each fragment?

Options: 1) Yes or 2) No

30. Consider the area where you practice most often. What is your degree of trust in the team responsible for providing the anatomopathological reports?

Options on a scale of 0 to 10; where 0 indicates you do not trust the team and 10 indicates you totally trust the team.

31. Consider the area where you practice most often. Do patients have easy access to magnetic resonance imaging (MRI)?

Options: 1) No, 2) Multiparametric MRI 1.5 Tesla, 3) Multiparametric MRI 3.0 Tesla, and/or 4) Pelvic (unspecified)

32. Who performs the prostate biopsy?

Options: 1) It is performed at my service, by a urologist; 2) It is performed at my service, by a radiologist; 3) I do it myself; and 4) It is outsourced.

33. In your service, what access is used in prostate biopsy?

Options: 1) Transrectal or 2) Perineal

34. Consider the area where you practice most often. What is the number of cores retrieved by biopsy?

Options: 1) Sextant (6 fragments), 2) Sextant (12 fragments, with 2 from each area), 3) 12 distinct fragments from the peripheral zone, 4) 12 distinct fragments from the peripheral zone + 2 to 4 fragments from the transition zone, 5) 12 distinct fragments from the peripheral zone + 2 to 4 fragments from the transition zone + additional fragments from the suspicious area (nodule on ultrasound or MRI), or 6) Other (please specify)

35. Do you have access to biopsy with image fusion?

Options: 1) No; 2) Yes, with real-time software fusion; or 3) Yes, with cognitive fusion

36. Consider the area where you practice most often. What is your degree of trust in the team responsible for performing prostate biopsies?

Options on a scale of 0 to 10; where 0 indicates you do not trust the team and 10 indicates you totally trust the team.

37. How long does it take for the patient to undergo the biopsy?

38. After the inclusion of a patient in an active surveillance protocol, do you perform a confirmatory biopsy?

Options: 1) No; 2) Yes, in 3 months; 3) Yes, between 3 and 6 months; or 4) Yes, between 6 and 12 months.

39. How often do you evaluate PSA levels during the first 5 years?

Options: 1) Quarterly, 2) Semiannual, 3) Quarterly in the first two years and semi-annual between the 2nd and 5th year, or 4) Annual.

40. How often do you perform a digital rectal exam?

Options: 1) I do not use it routinely, 2) Quarterly, 3) Quarterly in the first two years and half-yearly between the 2nd and 5th year, 4) Half-yearly measure, or 5) Annually.

41. In addition to confirmatory biopsy, how often do you perform new biopsies during the follow-up of patients under active surveillance?

Options: 1) Annual, 2) Biennial, 3) If the PSA level increases, 4) If imaging exam findings worsen, or 5) Other (please specify)

42. In addition to ultrasound for biopsy, do you use other imaging tests?

Options: 1) No, 2) MRI occasionally, or 3) MRI periodically

43. Which of the following options do you use to recommend conversion from active surveillance to definitive treatment? (More than one answer is acceptable).

Options: 1) Patient´s preference, 2) Worsened findings in rectal examination (worsening of clinical staging), 3) single rise in PSA level (PSA >10 ng/mL), 4) Two or more consecutive increases in PSA levels (PSA >10 ng/mL), 5) PSA doubling time < 3 years, 6) Rising PSA density (>15%), 7) Gleason score upgrading to 7 (3+4), 8) Gleason score upgrading to 7 (3+4) or greater on re-biopsy, 9) increased tumour volume on imaging (MRI), 10) Higher number of positive cores on re-biopsy (>1/3), and/or 11) tumour involvement > 50% in at least one core on re-biopsy

44. Do you feel safe indicating active surveillance for patients in the place where you primarily practice?

Options on a scale of 0 to 10; where 0 indicates you do not feel safe indicating and 10 indicates you totally feel safe on indicating active surveillance for patients in the place where you primarily practice.

45. For what reasons would you not indicate active surveillance in a 65-year-old patient, without comorbidities, potent and continent, diagnosed with low- or very-low-risk PCa?

Options: 1) I do not know enough about this type of treatment to properly follow-up the patients; 2) I believe that active surveillance is an inappropriate approach for patients with prostate cancer, even at low or very low risk; 3) morbidity associated with multiple biopsies; 4) the patient does not accept the active surveillance and instead requests active treatment; 5) I would like to indicate, but the biopsy performed is inadequate; 6) I would like to indicate, but I do not trust the team responsible for performing the biopsy; 7) I would like to indicate, but the histopathological report does not provide the necessary data; 8) I would like to indicate, but I do not trust the team responsible for the histopathological evaluation; or 9) I would like to indicate, but I cannot schedule laboratory tests and/or biopsy and/or outpatient return with the appropriate interval.
